# A Pvs25 mRNA vaccine induces complete and durable transmission-blocking immunity to *Plasmodium vivax*

**DOI:** 10.1038/s41541-023-00786-9

**Published:** 2023-12-14

**Authors:** Nawapol Kunkeaw, Wang Nguitragool, Eizo Takashima, Niwat Kangwanrangsan, Hiromi Muramatsu, Mayumi Tachibana, Tomoko Ishino, Paulo J. C. Lin, Ying K. Tam, Sathit Pichyangkul, Takafumi Tsuboi, Norbert Pardi, Jetsumon Sattabongkot

**Affiliations:** 1 Mahidol Vivax Research Unit, Faculty of Tropical Medicine, Mahidol University, Bangkok, Thailand; 2 Department of Molecular Tropical Medicine and Genetics, Faculty of Tropical Medicine, Mahidol University, Bangkok, Thailand; 3 Division of Malaria Research, Proteo-Science Center, Ehime University, Matsuyama, Japan; 4 Department of Pathobiology, Faculty of Science, Mahidol University, Bangkok, Thailand; 5 Department of Microbiology, Perelman School of Medicine, University of Pennsylvania, Philadelphia, PA USA; 6 Division of Molecular Parasitology, Proteo-Science Center, Ehime University, Toon, Japan; 7 Department of Parasitology and Tropical Medicine, Graduate School of Medical and Dental Sciences, Tokyo Medical and Dental University, Tokyo, Japan; 8 Acuitas Therapeutics, Vancouver, BC V6T 1Z3 Canada

**Keywords:** RNA vaccines, Malaria

## Abstract

*Plasmodium vivax* (*P. vivax*) is the major malaria parasite outside of Africa and no vaccine is available against it. A vaccine that interrupts parasite transmission (transmission-blocking vaccine, TBV) is considered highly desirable to reduce the spread of *P. vivax* and to accelerate its elimination. However, the development of a TBV against this pathogen has been hampered by the inability to culture the parasite as well as the low immunogenicity of the vaccines developed to date. Pvs25 is the most advanced TBV antigen candidate for *P. vivax*. However, in previous phase I clinical trials, TBV vaccines based on Pvs25 yielded low antibody responses or had unacceptable safety profiles. As the nucleoside-modified mRNA–lipid nanoparticle (mRNA–LNP) vaccine platform proved to be safe and effective in humans, we generated and tested mRNA–LNP vaccines encoding several versions of Pvs25 in mice. We found that in a prime-boost vaccination schedule, all Pvs25 mRNA–LNP vaccines elicited robust antigen-specific antibody responses. Furthermore, when compared with a Pvs25 recombinant protein vaccine formulated with Montanide ISA-51 adjuvant, the full-length Pvs25 mRNA–LNP vaccine induced a stronger and longer-lasting functional immunity. Seven months after the second vaccination, vaccine-induced antibodies retained the ability to fully block *P. vivax* transmission in direct membrane feeding assays, whereas the blocking activity induced by the protein/ISA-51 vaccine dropped significantly. Taken together, we report on mRNA vaccines targeting *P. vivax* and demonstrate that Pvs25 mRNA–LNP outperformed an adjuvanted Pvs25 protein vaccine suggesting that it is a promising candidate for further testing in non-human primates.

## Introduction

Over 3 billion people are currently at risk of *Plasmodium vivax* malaria and no approved vaccine is available against this disease^1^. In many countries, *P. vivax* malaria is a major public health problem, causing severe illness and economic hardships in remote populations. A growing number of studies have indicated severity, complications, and deaths due to *P. vivax* malaria in pregnant women and small children^2,3^. Compared to *Plasmodium falciparum*-caused disease, *P. vivax* malaria is harder to control and eliminate due to its ability to lie dormant as a hypnozoite in the human liver for months to years before it reactivates^4^ and cause a relapse of blood-stage infection. Additionally, the parasite has faster sexual development and higher mosquito infectivity compared to *P. falciparum*^5,6^, resuming its efficient capacity to transmit to new hosts. Current drug choices for killing hypnozoites are limited to 8-aminoquinolines, primaquine, and tafenoquine, but these drugs can cause acute hemolysis in people with glucose-6-phosphate dehydrogenase (G6PD) deficiency; therefore, they are contraindicated in patients with G6PD deficiency and during pregnancy and breastfeeding and are not recommended in children under the age of six months^7^. Therefore, a safe and effective vaccine would be vital for the elimination of *P. vivax*^8^.

Malaria vaccines can be categorized into pre-erythrocytic vaccines, which target sporozoites or liver-stage parasites, blood-stage vaccines, which target merozoites or infected erythrocytes, and transmission-blocking vaccines (TBVs) which target gametocytes and mosquito-stage parasites. Today, the most advanced malaria vaccines target the pre-erythrocytic stage of *P. falciparum*^9,10^, but several blood-stage and TBV candidates are in the development pipeline, including a few for *P. vivax*^11^. The development of new malaria vaccines with at least 75% efficacy and TBVs was proposed in the renewed Malaria Vaccine Technology Roadmap to 2030^12^. The advantage of TBVs over pre-erythrocytic and blood-stage vaccines is that the TBV candidates tend to be more conserved^13^, presumably due to lower exposure to human immunity^14,15^. Because TBVs operate on a small number of transmission-stage parasites in mosquitoes, there could be less chance of developing escape mutations and establishing infection^12^. In addition, because transmission-blocking immunity is primarily antibody-mediated^16^, assessing and tracking vaccine efficacy are more straightforward for TBVs than for other types of malaria vaccines. Despite these advantages, only a few protein targets have been considered for *P. vivax* TBVs. In fact, all *P. vivax* TBV antigens examined up to now (Pvs25, Pvs28, Pvs230, Pvs47, Pvs48/45, and PvHAP2) are orthologs of known *P. falciparum* vaccine targets^8,17^. The *P. vivax* ookinete surface protein Pvs25, which is abundantly expressed on the surface of zygotes/ookinetes and essential for the survival of ookinetes in the mosquito midgut^18^, is the most advanced antigen for *P. vivax* TBVs^12^. Vaccine-induced Pvs25-specific antibodies are expected to bind their ligand expressed on the parasite cell surface and inhibit parasite development in the mosquito^19^. Pvs25-based vaccines have been shown to induce complete transmission-blocking immunity in animal models^19–21^ and were evaluated in phase I clinical trials^22,23^. Unfortunately, the vaccine candidates, Pvs25 recombinant protein adjuvanted with Alhydrogel or Montanide ISA-51, did not progress further due to the low immunogenicity^22^ or high reactogenicity^23^ of these vaccines, respectively.

In recent years, various forms of mRNA-based vaccines have proven to be highly effective against infectious diseases both in preclinical and clinical studies^24^. One of the most promising vaccine platforms comprises nucleoside-modified mRNA encapsulated in lipid nanoparticles (LNPs)^25^. Nucleoside-modified mRNA–LNP vaccines developed by Pfizer-BioNTech and Moderna have demonstrated worldwide safety and effectiveness in curbing Coronavirus Diseases 2019 (COVID-19) in the general population^26^. The nucleoside-modified mRNA–LNP vaccine platform has the unique ability to potently induce antigen-specific germinal center B and T cell responses^27–29^ that are critical for the generation of protective and durable neutralizing antibodies both in preclinical models^30–32^ and humans^33,34^. Recently, studies have revealed that nucleoside-modified mRNA–LNP vaccines can induce robust immune responses against specific targets of each stage of *P. falciparum*’s life cycle^35–37^. These antigens included the circumsporozoite protein (PfCSP)^36,37^, the glutamic-acid-rich protein (PfGARP)^35^, and the Pfs25 protein^37^, which are vaccine targets of the pre-erythocytic, blood-, and transmission-stage pathogen, respectively. We hypothesized that the nucleoside-modified mRNA–LNP platform could be used to design and generate potent Pvs25 vaccines for *P. vivax*.

## Results

### Design and in vitro characterization of nucleoside-modified Pvs25 mRNA–LNPs

Four nucleoside-modified Pvs25 mRNAs were designed based on the sequence of the Pvs25 gene from the reference *P. vivax* strain Sal I. Two constructs (Pvs25A and Pvs25A I130T) express Pvs25 with wildtype signal peptide without the C-terminal glycosylphosphatidylinositol (GPI) anchor, which is essential for the cell surface localization of the parasite^38^. The Pvs25A I130T construct contains the I130T amino acid substitution that is predominant in the Asian *P. vivax* isolates^39^. The other two constructs (Pvs25F and Pvs25F I130T) encode the full-length Pvs25 with its C-terminal GPI anchor. Pvs25F is the wildtype (Sal I) sequence and Pvs25F I130T contains the I130T mutation.

**Fig. 1:**
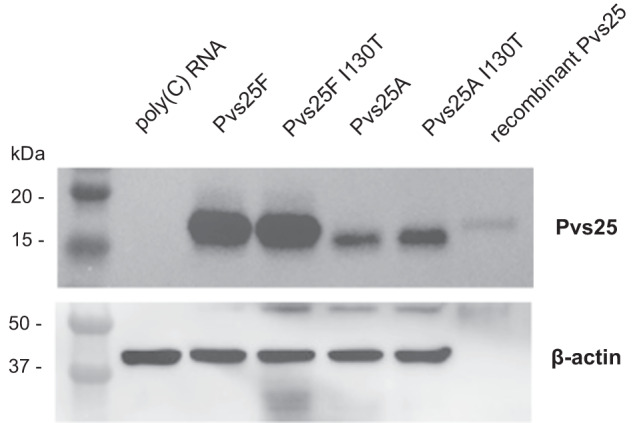
Protein expression from the nucleoside-modified Pvs25 mRNA–LNPs in vitro. HEK293 cells were transfected with the four different Pvs25 nucleoside-modified mRNA–LNPs or control poly(C) RNA–LNP, and protein production from each construct was examined in cell lysates by Western blotting using Pvs25-specific antibodies. Five nanograms of Pvs25 recombinant protein were used as a positive control. A β-actin-specific antibody was used as a loading control antibody. Two independent experiments were performed. All blots of gels derived from the same experiment and were processed in parallel.

Pvs25 mRNA–LNPs were produced and transfected into human embryonic kidney (HEK) 293 cells to assess protein production from the transfected mRNAs. Western blot analysis revealed protein production from each Pvs25 mRNA (Fig. 1). The levels of Pvs25 protein production from the two full-length constructs (Pvs25F and Pvs25F I130T) were higher than those from the truncated constructs (Pvs25A and Pvs25A I130T).

### Pvs25 mRNA–LNP vaccines induce robust antibody responses in mice

**Fig. 2:**
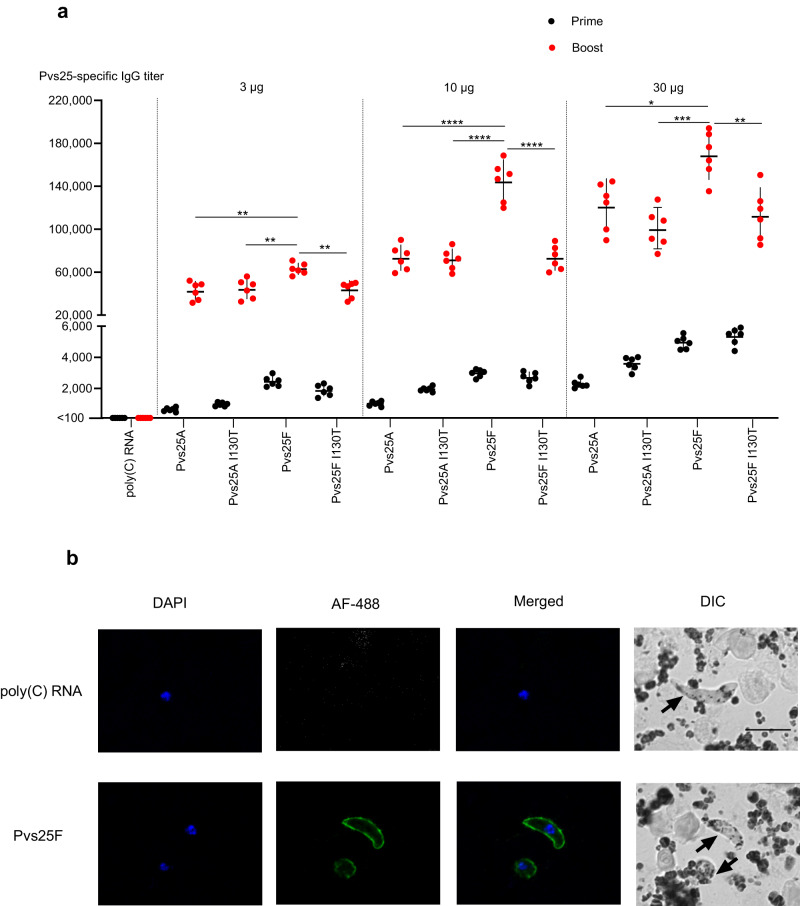
Nucleoside-modified Pvs25 mRNA–LNPs induce antigen-specific antibodies that recognize the native antigen. BALB/c mice received two doses of 3 µg, 10 µg, or 30 µg of Pvs25 mRNA–LNPs via intramuscular injection with a 4-week interval. Control mice received 30 µg of poly(C) RNA–LNP. **a** Pvs25-specific antibody responses were determined by endpoint dilution ELISA 4 weeks after the prime and 4 weeks after the boost and reported as the geometric mean titers (GMT) with 95% confidence intervals (CI). As we could not fit a curve for the control samples due to the low levels of antibodies, the endpoint titers were arbitrarily set to <100. n = 6, and each symbol represents one animal. Statistical analyses: Two-tailed t-test to compare prime vs. boost values (P < 0.0001 for all comparisons); One-way ANOVA with Dunnett’s post hoc test to compare, at each dose, the performance of different constructs to that of Pvs25F (* P < 0.05, ** P < 0.01, *** P < 0.001, **** P < 0.0001). **b** Pooled serum from animals immunized with 30 µg of Pvs25F mRNA–LNP or poly(C) RNA–LNP was used to assess reactivity to the native antigen on the surface of *P. vivax* ookinetes by immunofluorescence assay. Panels in the first column from the left show nuclear staining with DAPI, panels in the second column show staining with Alexa Fluor 488-conjugated anti-mouse IgG secondary antibody (AF-488), panels in the third column show the merged images, and panels in the fourth column show Differential Interference Contrast (DIC) images. Three independent experiments were performed using two different clinical *P. vivax* isolates and similar data were obtained (representative images obtained from one isolate are shown here). The scale bar indicates 10 µm.

Next, the Pvs25 mRNA–LNP vaccines were administered to BALB/c mice following a prime-boost schedule (week 0 and 4) via intramuscular injection using three different doses (3, 10, or 30 µg), and serum from each animal was collected 4 weeks after each immunization to determine the level of anti-Pvs25 antibody by ELISA (Fig. 2a). Mice receiving 30 µg of poly(C) RNA–LNP were used as a negative control. At 4 weeks after the first (prime) vaccination, the Pvs25-specific IgG induced by mRNA–LNPs was detectable with a geometric mean of reciprocal titer (GMT) value between 630–5300. After the booster dose, the antibody levels rose significantly reaching a GMT between 42,000–169,000 (Fig. 2a). At each dose, the Pvs25F mRNA–LNP outperformed other formulations. The pooled sera obtained from the Pvs25F mRNA–LNP-immunized mice 4 weeks after the boost recognized the native antigen on the surface of the ookinete (a midgut stage of the parasite) by immunofluorescence assay (IFA) (Fig. 2b). Antibodies induced by the other three mRNA–LNPs similarly recognized the native Pvs25 antigen by IFA (Supplementary Fig. 2). In contrast, the pooled sera from the control mice did not interact with the parasite, demonstrating that the Pvs25 mRNA–LNP vaccine induced strong antigen-specific antibody responses.

### Pvs25 mRNA–LNP vaccines induce antibodies with transmission-blocking activity

**Fig. 3:**
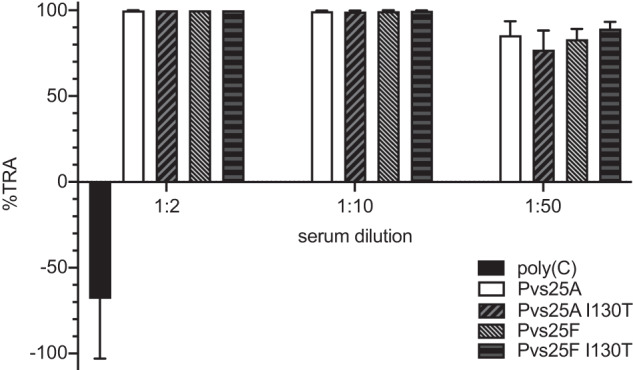
Functional activity of antisera induced by Pvs25 mRNA–LNP vaccines. Direct membrane feeding assay (DMFA) was performed to evaluate transmission-reducing activity (TRA) of immunized mouse antisera at different dilutions. Lab-reared uninfected mosquitoes were fed on *P. vivax* malaria patient blood (*n* = 5 patients) suspended with pooled serum obtained from mice (*n* = 6) immunized with 30 µg Pvs25 mRNA–LNP or 30 µg control poly(C) RNA–LNP. Mouse serum was diluted in human AB serum at the specified dilution factor. TRA represents the % reduction in the mean oocyst density for each test serum relative to the human AB serum. Error bars represent SEM. TRA was invariant across the four Pvs25 constructs (one-way ANOVA, *P* = 0.726; at 1:50 dilution).

The efficacy of the induced mouse antisera against *P. vivax* transmission was evaluated by direct membrane feeding assay (DMFA) in which blood samples from *P. vivax*-infected patients were fed to lab-reared *Anopheles dirus* mosquitoes. For each experiment, infected red blood cells were suspended in the immune serum obtained from mice that received 30 µg of each of the four Pvs25 mRNA–LNPs and serially diluted in human blood-type AB serum (serum from a naïve AB blood. The control immune serum, obtained from mice that received 30 µg of poly(C) RNA–LNP, was diluted at 1:2 in human AB serum. Mosquitoes were dissected 7 days after feeding and the number of *P. vivax* oocysts was determined by light microscopy. The transmission-reducing activity (TRA) of the elicited antibodies was defined as the percent reduction in the average oocyst number per mosquito by each serum sample (Fig. 3). In contrast to the sera from control animals that enhanced the oocyst number by ~1.7-fold, all samples from Pvs25 mRNA–LNP-immunized mice exhibited complete (100%) transmission-blocking activity at 1:2 dilution. TRA of these sera remained nearly complete at 1:10 dilution and were ~80% at 1:50 dilution. No statistically significant difference was detected in the TRAs of the four Pvs25 constructs at each dilution.

### Antibody and cellular responses following homologous and heterologous (protein and mRNA) prime-boost immunization

The Pvs25F mRNA–LNP vaccine at 10 µg was selected for subsequent experiments because it elicited comparably high Pvs25-specific IgG titers to the 30 µg vaccine dose. We compared the immunogenicity and kinetics of the immune response of four different immunization regimens: (a) Pvs25F mRNA–LNP homologous prime-boost vaccination, (b) recombinant Pvs25 (rPvs25) protein (with ISA-51 adjuvant) homologous prime-boost vaccination, (c) Pvs25F mRNA–LNP/rPvs25 protein heterologous vaccination, and (d) rPvs25 protein/Pvs25F mRNA–LNP heterologous vaccination. Mice were randomly assigned into eight groups (*n* = 17 mice per group). In four vaccination groups, mice were vaccinated with homologous and heterologous prime-boost regimens (10 µg mRNA–LNP or 10 µg adjuvanted recombinant protein vaccine, 4 weeks between the prime and boost). In the other 4 groups, 10 µg poly(C) RNA–LNP or Montanide ISA-51 VG was administered as negative controls for each vaccination group.

**Fig. 4:**
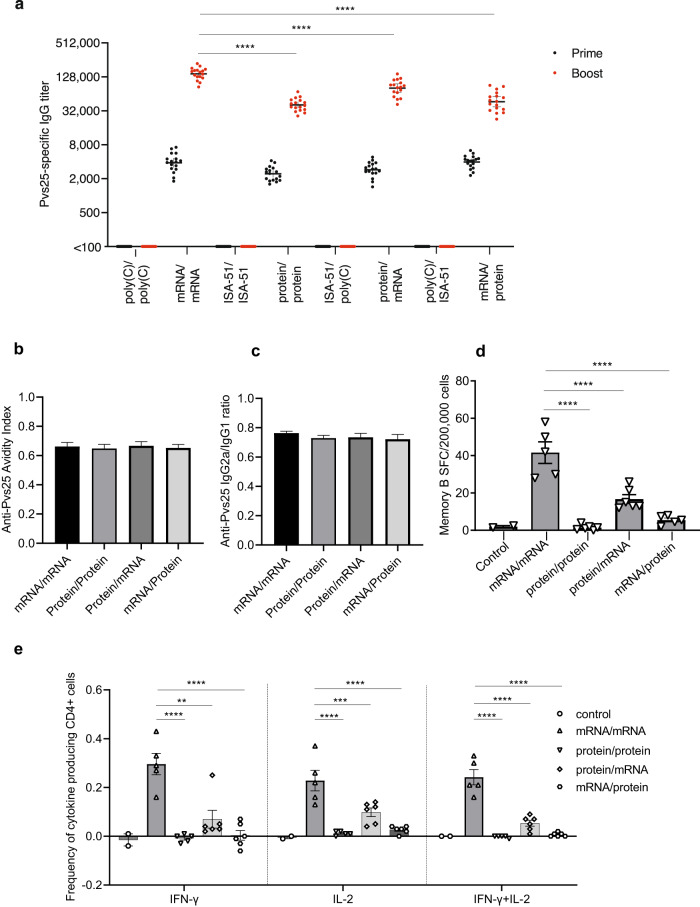
Antibody and cellular responses elicited by homologous and heterologous prime-boost vaccination. BALB/c mice were randomly assigned into eight groups (*n* = 17 per group). In four vaccination groups, mice were vaccinated with homologous and heterologous prime-boost regimens (permutations of 10 µg Pvs25F mRNA–LNP or 10 µg rPvs25 Protein-ISA51 VG). In the other 4 groups, 10 µg poly(C) RNA–LNP and Montanide ISA51 VG were administered as controls. Four weeks after the booster dose, eight mice from each group were terminated. Serum and spleen from each animal were collected to evaluate antibody and cellular responses. **a** Pvs25-specific IgG titers 4 weeks after the prime and 4 weeks after the boost vaccination were evaluated by endpoint dilution ELISA (*n* = 17 per group) and reported as GMT with 95% confidence intervals (CI). **b** Avidity indices of antibodies 4 weeks post boost assessed by ELISA (*n* = 8 per group). The avidity index (AI) of each mouse was calculated as the ratio of the areas under the curve obtained in the ELISA graphic (OD vs. log of dilution) with and without the denaturant. **c** Measurements of Pvs25-specific IgG2a and IgG1 subclasses from individual mice (*n* = 8 per group), expressed as the IgG2a/IgG1 ratio. **d**, **e** Splenocytes were obtained 4 weeks post boost (*n* = 5–6 per group). Control animals (*n* = 2) were randomly selected from the 4 control mice. **d** The Pvs25-specific memory B cell response was measured by ELISpot assay. Data are reported as spot-forming cells (SFC) per 200,000 input cells. **e** Splenocytes were stimulated with a Pvs25-specific peptide pool to analyze the production of IFN-γ and IL-2 by CD4^+^ T cells. Error bars represent SEM. **a**, **d**, **e** Each symbol represents one animal. Statistical analyses: **a** Two-tailed *t*-test to compare prime vs. boost values (*P* < 0.0001 for all comparisons); one-way ANOVA with Dunnett’s post hoc test to compare antibody titers to that of mRNA/mRNA (****P < 0.0001). **b**, **c** One-way ANOVA with Bonferroni correction (*P* > 0.05, *n* = 8 per group). **d**, **e** One-way ANOVA with Dunnett’s post hoc test to compare values to that of mRNA/mRNA (**P < 0.01, ***P < 0.001, ****P < 0.0001).

Sera from the control groups had no detectable Pvs25-specific antibody response by ELISA (Fig. 4a). The mRNA/mRNA homologous prime-boost vaccination generated the strongest Pvs25-specific antibody response with GMT of ~140,000. The protein/protein homologous vaccination had a GMT of ~40,000. The protein/mRNA vaccination had a GMT of ~80,000 and the mRNA/protein vaccination yielded a GMT of ~46,000.

We further characterized the quality of Pvs25-specific antibodies. IgG1 and IgG2a subclasses expressed as the IgG2a/IgG1 ratio and antigen-antibody avidity index have been previously shown to correlate with the functional activity of TBVs for *P. falciparum*^40,41^. The IgG subclass patterns of all vaccination regimens were similar with the IgG2a/IgG1 ratio of ~0.75. The avidity indices were also similar across the groups (Fig. 4b, c).

Mouse splenocytes from the homologous and heterologous prime-boost experiments were evaluated for cellular immune responses. The mRNA/mRNA homologous vaccination elicited the strongest memory B cell response, whereas this was almost absent in the protein/protein homologous vaccination group (Fig. 4d). Likewise, the mRNA/mRNA homologous prime-boost vaccination induced the most robust Pvs25-specific CD4^+^ T cell responses as measured by IFN-γ and IL-2 production of CD4^+^ T cells, while the protein/protein vaccination barely induced T cell responses (Fig. 4e). The protein/mRNA heterologous vaccination gave positive but intermediate cellular responses while the mRNA/protein vaccination elicited very low cellular response similar to the protein/protein vaccination.

### Pvs25 mRNA vaccination elicits durable antibodies with potent malaria transmission-reducing activity

**Fig. 5:**
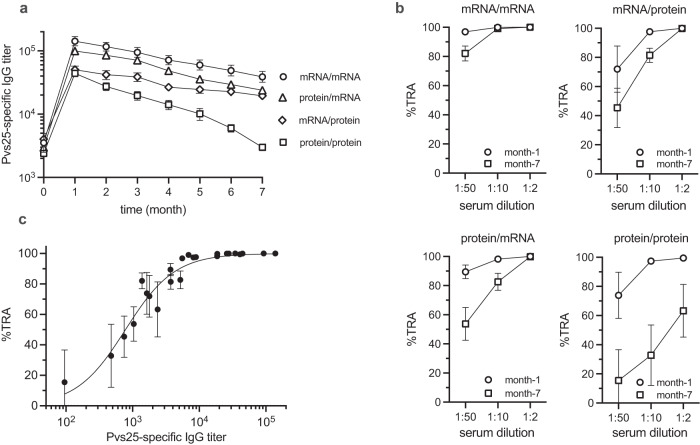
Antibody durability and functional response elicited by different prime-boost regimens. **a** Time courses of the Pvs25-specific antibody responses measured by endpoint dilution ELISA reported as GMT with 95% CI. Time (month) indicates the time after the boost vaccination. *n* = 8–9 mice per group. **b** TRA induced by the different immunization regimens at 1- and 7-month post boost vaccination. The experiment was conducted using four different clinical *P. vivax* isolates (*n* = 4). TRA was determined at 1:2, 1:10, and 1:50 serum dilutions. **c** Relationship between TRA (from (**b**)) and total Pvs-25 specific IgG (Spearman *ρ* = 0.963, *P* < 0.0001). Error bars represent SEM in panels (**b**) and (**c**). In **c**, data fit with Hill’s equation.

Pvs25-specific antibody levels of animals immunized in the homologous/heterologous prime-boost studies described above were followed monthly for 7 months post boost vaccination to assess the durability of antibody responses (Fig. 5a). The antibody levels peaked at 1-month post boost in all vaccination groups and declined over the subsequent months. In agreement with the previous results, GMTs of Pvs25-specific antibodies were highest in the mRNA/mRNA group and the lowest in the protein/protein group, with antibody response half-lives of 3.2 and 1.7 months, respectively. A similar pattern was observed when the TRA was followed over time; the TRA was the highest one month after boosting (Fig. 5b). At this time, all vaccination regimens rendered the full efficacy (100% TRA) at serum dilution 1:2. However, at the same 1:2 dilution, the TRA of protein/protein vaccination declined to 63% whereas the other three vaccination strategies retained high efficacy >99% at 7 months post boost. As serum was diluted further to 1:10 and 1:50, TRA declined as expected.

When the TRA data across all immunization regimens are pooled, there is a clear dose–response relationship between TRA and total IgG (Fig. 5c). The half-maximal inhibition concentration (IC_50_) of Pvs25-specific total IgG was 781 (CI_95_: 564–1003) reciprocal titer unit.

## Discussion

In this study, we evaluated Pvs25-based vaccines utilizing the nucleoside-modified mRNA–LNP technology, which performed very well during the COVID-19 pandemic^26^. The rationale for selecting Pvs25 as the vaccine antigen candidate was that this protein has already been validated as an excellent target for *P. vivax* TBVs. Pvs25 protein-subunit vaccines have been shown to induce complete transmission-blocking immunity in animals^19–21^. The failure of Pvs25 protein-subunit vaccines in previous clinical trials^22,23^ was due to their inability to elicit potent antibody responses in humans with an acceptable safety profile. The recent success of the nucleoside-modified mRNA–LNP technology suggests that this novel platform has the potential to overcome these challenges. This study is the first attempt to apply the mRNA–LNP technology for Pvs25-based TBVs. Importantly, we demonstrate that Pvs25 mRNA–LNP vaccines outperform an adjuvanted Pvs25 protein vaccine and yield very promising results in mice.

To identify the optimal vaccine antigen, four different Pvs25 mRNA constructs were produced and evaluated for their ability to induce robust antibody responses in mice. Previous studies have suggested that the presence of a GPI anchor can enhance the immune response to Pfs25 vaccines^42,43^. Our results do not always confirm these findings with Pvs25 mRNA–LNP vaccines. The inclusion of the GPI anchor as part of the full-length construct appeared to strengthen the immune response of the wild-type constructs (Pvs25F vs. Pvs25A), but not the I130T constructs (Pvs25F I130T vs. Pvs25A I130T). Thus the role of the GPI anchor for the Pvs25 mRNA–LNP vaccines remains unclear. The point mutation I130T predominant in Asian *P. vivax* isolates also did not have a discernable impact on vaccine-induced TRA; thus, it is unlikely to be a concern for Pvs25 TBVs. We found that the full-length construct Pvs25F elicited the strongest antibody responses at all tested doses. The construct was subsequently chosen to benchmark against the Pvs25 protein/ISA-51 vaccine, formulated after a previous vaccine candidate that reached evaluation in clinical trial^23^. Of note, findings in cell transfection studies were different from mouse vaccination studies: both Pvs25F mRNAs outperformed Pvs25A mRNAs in protein production in HEK293T cells, but the Pvs25F I130T-based vaccine was not superior compared to the Pvs25A vaccines in mice. These data suggest that there is no clear association between the in vitro HEK293T protein expression levels and the antibody responses induced by the Pvs25 mRNA–LNP vaccines in mice. Therefore, in vitro protein expression in this cancer cell line is not a reliable predictor for vaccine immunogenicity.

In the homologous prime-boost immunization schedule, the Pvs25 mRNA–LNP vaccine mounted a stronger peak antibody response than the protein/ISA-51 vaccine. The antibody response to the mRNA vaccine also had a longer half-life (3.2 months) than to protein vaccination (1.7 months). Because both the magnitude and half-life of the antibody response contribute to the durability of immunity, the time for the antibody response to decline from its peak value to IC_50_ (reciprocal titer = 781) was calculated. With the assumption of constant exponential decline, the antibody induced by the protein/protein vaccination would fall from its peak value to IC_50_ in 9.5 months. In contrast, the mRNA/mRNA vaccine-induced antibody would last for 24 months before reaching the IC_50_ (Supplementary Fig. 3). Thus, by this measure, antibodies elicited by the mRNA vaccine were 2.5 times more durable than those induced by the protein vaccine. The durability of vaccine-induced antibody response is critical for a post-fertilization TBV target such as Pvs25 because this antigen is present only in the transmission-stage parasite inside the mosquito midgut and, therefore, there is little chance for immunological boosting through natural infection^14^. It is important to note that the two-dose schedule at 10 µg may not be optimal for the protein/ISA-51 vaccine; it was chosen to provide an appropriate comparator for the mRNA–LNP vaccine. The lack of the GPI anchor in the recombinant protein vaccine may be suboptimal for immune response activation; thus, the comparison between the mRNA–LNP (containing the GPI anchor) and protein/ISA-51 platforms was not direct. The transmission-reducing antibody response against Pfs25, the *P. falciparum* ortholog of Pvs25, was extensively studied in multiple experimental animals and humans. Based on this accumulated evidence, the required concentration of Pfs25-specific IgGs to inhibit 50% of oocyst development (IC_50_) in the blood were 15.9, 4.2, 41.2, and 85.6 µg/mL for mice, rabbits, monkeys, and humans, respectively, and the required anti-Pfs25 IgG concentration in humans is 5 times higher than that in mouse^44^. Based on our data with the Pvs25 mRNA–LNP vaccine in mice, further testing in non-human primates is warranted.

The more durable antibody responses induced by the mRNA–LNP vaccine compared to the protein/ISA-51 vaccine are consistent with the higher number of splenic cytokine-producing CD4^+^ T cells and antigen-specific memory B cells. The magnitude and durability of antibody response induced by a vaccine are shaped by its ability to induce T follicular helper (Tfh) cells, a subset of CD4^+^ T cells, whose formation takes place in the germinal center (GC) in the secondary lymphoid organs^45^. The great ability of nucleoside-modified mRNA–LNP vaccination to elicit GC B and Tfh cells has been revealed in animal models^27,29^ as well as in humans immunized with SARS-CoV-2 mRNA vaccines^46,47^. Accordingly, the nucleoside-modified mRNA–LNP platform is likely the primary reason for the improvement in vaccine efficacy over the protein/ISA-51 vaccine (as shown in other comparative mRNA/protein vaccine studies)^48^. In addition, the malaria parasite GPI enhanced the antibody responses reported in Pfs25 vaccine studies^42^. Therefore, the use of an mRNA encoding the full-length (GPI-containing) Pvs25 protein (Pvs25F) may also contribute to this enhancement in its performance compared to the rPvs25 protein antigen.

The progress in developing *P. vivax* TBVs has lagged behind that of *P. falciparum* TBVs. Strategies to accelerate TBV research for *P. vivax* are to enhance the immunogenicity of the existing antigen candidates through potent vaccine platforms or to discover novel vaccine antigens^12,14^. Recently, the application of the nucleoside-modified mRNA–LNP platform for targeting *P. falciparum* malaria antigens has been shown to be highly effective in eliciting protective immunity^35–37^. An mRNA–LNP expressing PfGARP stimulated the production of antibodies capable of attenuating severe malaria in nonhuman primates^35^. Nucleoside-modified mRNA vaccines encoding PfCSP were found to be immunogenic and protective in mice^36^. Immunization with Pfs25 and PfCSP mRNA–LNPs individually or as a combination induced a potent immune response with high effectiveness in reducing *P. falciparum* CSP transgenic *P. berghei* parasites infection in mice and parasite transmission in the mosquito vector^37^. Here, our results demonstrated that a nucleoside-modified mRNA–LNP vaccine encoding Pvs25 can induce cellular as well as durable antigen-specific antibody responses with potent parasite transmission-blocking activity in mice, providing strong support for further preclinical testing in nonhuman primates. Given the positive results, it seems worthwhile to apply the mRNA–LNP platform to other *P. vivax* vaccine antigen candidates^49^ and potentially evaluate multivalent *P. vivax* mRNA–LNP vaccine formulations as successfully shown for influenza virus^50–53^. The platform has the potential to overcome the challenges of poor immunogenicity, strong reactogenicity, low potency, and short-lived immunity.

## Methods

### Design of nucleoside-modified Pvs25 mRNA–LNPs

Four nucleoside-modified Pvs25 mRNA constructs were designed based on the sequence of the Pvs25 gene from the reference *P. vivax* strain Sal I (PVX_111175). Pvs25A constructs were designed with wildtype signal peptide without the C-terminal GPI anchor, which is essential for parasite cell surface localization^38^. Pvs25A has the wildtype sequence, and Pvs25A I130T contains the I130T substitution predominant in the Asian *P. vivax* isolates^39^. The other two full-length Pvs25 constructs were as follows. Pvs25F encodes the full-length sequence of the Pvs25 gene from Sal I with wild-type signal peptide. Pvs25F I130T construct contains the full-length sequence of Pvs25 with the I130T mutation. Polycytidine (Poly(C)) RNA–LNP was used as a negative control construct.

### Production of nucleoside-modified Pvs25 mRNA–LNPs

mRNAs were in vitro transcribed using T7 RNA polymerase (Megascript; Ambion) on linearized plasmids encoding mammalian codon-optimized Pvs25. mRNAs were generated to contain 101 nucleotide-long poly(A) tails and modified by replacing uridine-5′-triphosphate with m1Ψ-5′-triphosphate (TriLink BioTechnologies). mRNA capping was performed alongside transcription through the addition of a trinucleotide cap1 analog, CleanCap (TriLink), and mRNA was purified with cellulose-based purification^54^. The size and integrity of mRNAs were analyzed on an agarose gel before storing at −20 °C. Purified mRNAs and poly(C) RNA (Sigma) were LNP-encapsulated using a self-assembly process where a mixture of an ionizable cationic lipid, phosphatidylcholine, cholesterol, and polyethylene glycol-lipid in ethanol was rapidly combined with an aqueous solution containing mRNA at acidic pH. The ionizable cationic lipid (pKa in the range of 6.0–6.5, proprietary to Acuitas Therapeutics) and LNP composition are described in the patent application WO 2017/004143. The average hydrodynamic diameter was ∼80 nM with a polydispersity index of 0.02–0.06 as measured by dynamic light scattering using a Zetasizer Nano ZS (Malvern Instruments Ltd.) and an encapsulation efficiency of ∼95% was achieved as determined using a Ribogreen assay.

### Production of recombinant Pvs25 protein (rPvs25)

Recombinant Pvs25 protein (rPvs25) was expressed using a wheat germ cell-free expression system (WGCFS: CellFree Sciences, Matsuyama, Japan). DNA sequences corresponding to the amino acid (aa) region A23-L195 of Pvs25 (PVX_111175) without the signal peptide and the GPI-anchor sequences and with a C-terminal hexahistidine tag (his-tag) were cloned into wheat germ cell-free expression vector pEU (CellFree Sciences). The Pvs25 recombinant protein (rPvs25) was expressed using the WGCFS and Ni-affinity purified.

### Cell transfection studies

Protein production from the Pvs25 mRNA–LNPs was confirmed by transfecting human embryonic kidney (HEK) 293 cells with Pvs25 mRNA–LNPs. The cells were seeded at 300,000 cells/well of a 24-well plate. After 16-hour incubation, the cells were transfected with 2 µg/well mRNA–LNP. Transfected cells were collected 18 h post-transfection, cell lysates were prepared, and Western blotting was performed to confirm protein production from the mRNA–LNP constructs.

### Western blotting

The samples were solubilized in NuPAGE™ LDS sample buffer (Invitrogen NP0008) and separated on a 4–15% precast polyacrylamide Criterion TGX gel (Bio-Rad) for 1 h at 100 V. Semi-dry Transfer to polyvinylidene fluoride (PVDF) membrane was performed at 25 V for 1 h. The membrane was blocked in 5% (w/v) skim milk in Tris Buffered Saline with Tween-20 (TBST) for 1 h, and then, the membrane was incubated with the pooled mouse antiserum immunized with recombinant rPvs25 protein (1:20,000) and mouse anti-β-actin antibodies (1:10,000 for the loading control; Abcam ab8226) in 5% (w/v) skim milk/TBST, washed three times, probed with horseradish peroxidase-conjugated anti-mouse IgG secondary antibodies (1:25,000; Merck Millipore 12–349), and developed using Immobilon Western chemiluminescent horseradish peroxidase (HRP) substrate (Merck Millipore WBKLS0500). The full blot images are available in Supplementary Fig. 1.

### Mouse immunization

All animal studies used 8-week-old female BALB/c mice (Nomura Siam International). Mice were randomly allocated to groups. For identification of the optimal mRNA construct, mice (*n* = 6) received two vaccinations of 3 µg, 10 µg, or 30 µg of Pvs25 mRNA–LNPs. A control mice group (*n* = 6) received two doses of 30 µg of poly(C) RNA–LNP. For comparison of mRNA and protein vaccines, four experimental groups (*n* = 17 per group) received homologous or heterologous prime-boost vaccination of 10 µg of a Pvs25 mRNA–LNPs or 10 µg rPvs25 Protein-ISA51 VG. Four control groups (*n* = 17 per group) were administered with 10 µg poly(C) RNA–LNP or Montanide ISA51 VG in a similar manner. Mice were immunized intramuscularly without anesthesia two times at weeks 0 and 4. The injection volume of vaccines was 100 μL. Prior to each immunization, 100 μL of blood was collected by the tail-clip method. Mice were euthanized with carbon dioxide (CO_2_) 4 weeks or 7 months after the last immunization. The euthanasia chamber was filled at a rate of 30–70% of the chamber volume per minute with CO_2_. Immediately after euthanasia, 0.5–1 mL of blood was collected by cardiac stick, and the spleen was removed for splenocyte preparation. Splenocytes were separated by mechanical homogenization. A cell suspension was filtered through a 70 μM cell strainer (SPL Life Sciences 93070). Red Blood Cells were removed by using lysing buffer (BD Biosciences 555899), then following a wash with RPMI 1640 medium (Thermo SCIENTIFIC 21870076). The animal protocol used in this study was approved by the Institutional Animal Care and Use Committee of the Faculty of Tropical Medicine, Mahidol University, and followed accordingly.

### Protein formulation with Montanide ISA 51 VG

Recombinant Pvs25 produced by WGCFS was formulated with Montanide ISA 51 VG (Seppic 36362Z) according to manufacturer instructions for immunization as an adjuvanted protein vaccine. 300 µg of recombinant Pvs25 was diluted in 1 mL of PBS and used to manufacture 2 mL of water-in-oil emulsion in a 50/50 volumic ratio (1 volume of Montanide ISA 51 VG for 1 volume of aqueous phase) according to manufacturer instruction. In total, 67 µL of the formulation (10 µg of recombinant Pvs25 protein) was used for each mouse immunization with a similar interval as the mRNA vaccination described above.

### Enzyme-linked immunosorbent assay

Endpoint dilution enzyme-linked immunosorbent assay (ELISA) was performed to determine Pvs25-specific antibody titers after vaccination. Briefly, the ELISA plates were coated with 100 μl of the 1 μg/mL of rPvs25 protein overnight at 4 °C and washed with PBS three times before blocking with 5% skim milk in PBS for 2 h at 37 °C. Two-fold serially diluted mouse sera (starting at 1:100) was added and incubated for 1 h at 37 °C. Plates were washed as above and incubated with HRP-conjugated anti-mouse IgG secondary antibodies (1:2,000; Merck Millipore 12–349) for 1 h at 37 °C. Following three times washing with PBS, ABTS (2,2’-Azino-bis [3-ethylbenzthiazoline-6-sulfonic acid]) (Merck Millipore ES004-500ML) was applied as a substrate. The enzyme reaction was stopped with 1% SDS and the absorbance at 415 nm (OD_415_) was read. The absorbance of serial dilution of an individual test serum was fitted to four four-parameter logistic (4 L) regression curves. The reciprocal of the dilution, giving an OD_415_ of 1, was designated as the endpoint for ELISA titers. IgG1 and IgG2a subtypes were profiled by ELISA similarly to the determination of antibody titers, except that HRP-conjugated anti-mouse IgG1 (Abcam ab97240) or IgG2a (Abcam ab97245) was applied instead.

Avidity ELISA: the diluted sera were assayed in the presence and absence of 1 M NH_4_SCN and incubated for 15 min at room temperature. The rest of the assay was performed as described above. The avidity index was then calculated as the ratio of the ODs under the denaturing condition divided by the ODs under the control condition.

### Immunofluorescence assay

Indirect IFA was performed to assess antibody reactivity against the native *P. vivax* Pvs25. The ookinete slides were prepared by dissecting mosquitoes fed with *P. vivax*-infected patient blood 16–20 h after feeding. The bloodmeal in a midgut was resuspended in 50 µl of PBS and 10 µL of the suspension was spotted on a multi-well slide glass (Thermo SCIENTIFIC ER-308B-CE24). After the air dry, the antigen slide was fixed with ice-cold acetone for 5 min, air-dried, and stored at –80˚C until use. The slide was blocked with 5%-milk PBS for 30 min at RT, then probed with mouse immune sera for 60 min at RT. After the three-times consecutive washing with PBS, the slide was stained with Alexa 488-conjugated anti-mouse antibody (Invitrogen A20181) and 4’,6-diamidino-2-phenylindole (DAPI) (1:1,000, Cell Signaling Technology 2350) for 30 min at RT. After the final wash, the slide was mounted in ProLong Antifade reagent (Invitrogen). The slides were observed by confocal scanning laser microscopy (LSM700, Zeiss).

### Assessment of transmission-blocking activity

The direct membrane feeding assay (DMFA) was utilized to evaluate the capacity of mouse antisera to block malaria parasite development in mosquitoes. Venous blood was collected, with written informed consent, from *P. vivax* volunteer patients who came to a malaria clinic in the Tha Song Yang district in the Tak province of northwestern Thailand. Single species infection with *P. vivax* was first identified by light microscopy and subsequently confirmed by nested PCR. Plasma was removed from the heparinized blood to obtain packed infected red blood cells. Mouse antisera were mixed with an equal volume of naïve human AB serum to achieve the 1:2 dilution mentioned elsewhere in the article. At higher dilutions (1:10 and 1:50), this 1:2 mixture was serially diluted 5-fold. The prepared mouse antisera–human AB serum mixture was added to infected red blood cells at a 1:1 v/v ratio. The mixture was applied to a membrane feeding apparatus kept at 37 °C to feed 100 lab-reared female *An. dirus* A mosquitoes for 30 min. Fully engorged mosquitoes were selected and maintained for a week in an insectary kept at 26 °C. For each test sample, 20 mosquitoes were dissected for light microscopic examination and oocysts developed in each mosquito were counted. Results are expressed as transmission-reducing activity (%TRA), which represents the percent reduction of the mean oocyst intensity per midgut of all mosquitoes dissected relative to the AB-serum control. Human subject research conducted as part of this study was reviewed and approved by the Ethics Committee of the Faculty of Tropical Medicine, Mahidol University (MUTM-2018-2016-05).

### Memory B cell response

Pvs25-specific memory B cell response was measured by ELISpot assay. Briefly, splenocytes were cultured in 48-well plates for 48–72 h in the presence of 1 μg/mL Resiquimod (R848) and 10 ng/mL IL-2 using Mouse memory B cell StimPack (Mabtech 3661-1). To enumerate Pvs25-specific antibody-secreting cells, stimulated cells were added into ELISpot plates (Merck Millipore MSIPS4W10) coating with 0.5 μg recombinant rPvs25 per well and cultured overnight. Plates were washed, followed by incubation with anti-mouse conjugated with biotin (Mabtech 3825-6-250), and incubated for 2 h at 37 °C. The plate was washed and incubated with streptavidin–alkaline phosphatase (Mabtech 3310-10-1000) for 1 h and developed by 5-bromo-4-chloro-3-indolyl phosphate-NBT-blue system (Mabtech 3650-10). The reaction was stopped by washing with tap water and air-dried. Spot-forming cells (SFC) were quantified by an automated plate reader (Cellular Technology Ltd.).

### T cell response

IFN-γ and IL-2 responses against Pvs25 in CD4^+^ T cells were measured by intracellular cytokine staining. Briefly, splenocytes were cultured for 18 h at 37 °C in 5% CO_2_ in 96 U-bottom wells in a volume of 200 µl (1 × 10^6^ cells) with a pool of 52 Pfs25 synthetic peptides (1 µg/mL). Fifty overlapping synthetic peptides (15 amino acids long with an 11 amino acid overlap) of Pfs25 (amino acids 1–219) were synthesized by GeneScript Biotech (Singapore). Peptides were supplied in the lyophilized form and estimated to be >80% pure. GolgiPlug (BD Biosciences 51-2301KZ) was added for the last 16 h to inhibit cytokine secretion. Then cells were washed and surface stained with anti-mouse CD3 (BioLegend 100306) and CD4 (BioLegend 100538) antibodies. Following surface staining, cells were washed in Fix/Perm buffer (BD Biosciences 51-2090KZ) and fixed using the Cytofix/Cytoperm kit (BD Biosciences 555028) according to the manufacturer’s instructions. Following fixation, the cells were washed in the Perm/wash buffer (BD Biosciences 51-2091KZ) and incubated with antibodies against IFN-γ (BioLegend 505826) and IL-2 (BioLegend 503808). An unstimulated sample was included as a negative control. Samples stimulated with 10 ng/mL phorbol myristate acetate (PMA) (Sigma-Aldrich SIA-I0634) and 250 ng/mL ionomycin (Sigma-Aldrich SIA-I0634) were used as positive controls. Finally, stained cells were analyzed by six-color flow cytometry (BD FACSCanto; BD Biosciences).

### Data analysis

Statistical analyses and non-linear curve fitting were performed using GraphPad Prism version 9. Specific statistical tests used are indicated in the main text next to each *P* value. The TRA-IgG dose response (Fig. 5c) was fitted with a two-parameter Hill’s equation yielding an IC_50_ estimate of 781 Pvs25-specific IgG titer.

Assuming a simple exponential decline, the half-life (*τ*) of each antibody decline was calculated from data in Fig. 5a as *τ* = log(2)/*β* where *β* is the slope of linear regression log(Ab) = *α* − *βt* over the period of 1–7 months. The time to reach IC_50_ from the maximal antibody (month 1) was calculated by solving for *t* in IC_50_ = Ab_max_2^−*t*/*τ*^ where IC_50_ was 781.

### Reporting summary

Further information on research design is available in the Nature Research Reporting Summary linked to this article.

### Supplementary information

Supplementary

Reporting Summary

## Data Availability

The datasets generated and/or analyzed during the current study are available from the corresponding authors upon reasonable request.
