# Author Correction: A Pvs25 mRNA vaccine induces complete and durable transmission-blocking immunity to *Plasmodium vivax*

**DOI:** 10.1038/s41541-024-00837-9

**Published:** 2024-02-19

**Authors:** Nawapol Kunkeaw, Wang Nguitragool, Eizo Takashima, Niwat Kangwanrangsan, Hiromi Muramatsu, Mayumi Tachibana, Tomoko Ishino, Paulo J. C. Lin, Ying K. Tam, Sathit Pichyangkul, Takafumi Tsuboi, Norbert Pardi, Jetsumon Sattabongkot

**Affiliations:** 1 Mahidol Vivax Research Unit, Faculty of Tropical Medicine, Mahidol University, Bangkok, Thailand; 2 Department of Molecular Tropical Medicine and Genetics, Faculty of Tropical Medicine, Mahidol University, Bangkok, Thailand; 3 Division of Malaria Research, Proteo-Science Center, Ehime University, Matsuyama, Japan; 4 Department of Pathobiology, Faculty of Science, Mahidol University, Bangkok, Thailand; 5 Department of Microbiology, Perelman School of Medicine, University of Pennsylvania, Philadelphia, PA USA; 6 Division of Molecular Parasitology, Proteo-Science Center, Ehime University, Toon, Japan; 7 Department of Parasitology and Tropical Medicine, Graduate School of Medical and Dental Sciences, Tokyo Medical and Dental University, Tokyo, Japan; 8 Acuitas Therapeutics, Vancouver, BC V6T 1Z3 Canada

**Keywords:** RNA vaccines, Malaria

Correction to: *npj Vaccines* 10.1038/s41541-023-00786-9, published online 14 December 2023

In the original version of this Article, an equal supervision statement was missing as well as a reference for cellulose-based mRNA purification. They have now been added to the HTML and PDF versions of the Article.

