# Supplementary material for: A Pvs25 mRNA vaccine induces complete and durable transmission-blocking immunity to Plasmodium vivax
Source: NPJ Vaccines. 2023 Dec 14;8:187. doi: 10.1038/s41541-023-00786-9 (PMC10719277; doi:10.1038/s41541-023-00786-9)
Supplement: Supplementary file 1 — Supplementary [file 41541_2023_786_MOESM1_ESM.pdf]

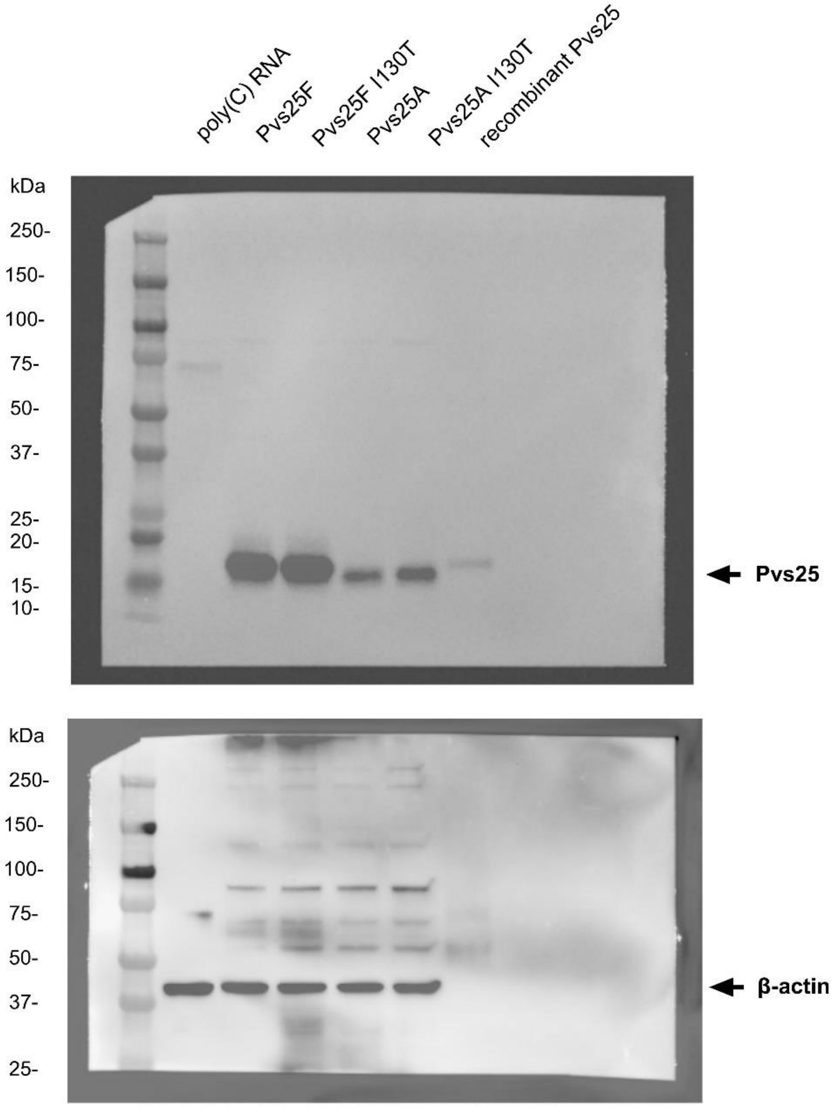

Supplementary Fig. 1 Immunoblot showing protein expression in HEK293T after transfecting with four different mRNA-LNPs. After polyacrylamide gel electrophoresis and the transfer of proteins to polyvinylidene fluoride (PVDF) membrane, the membrane was strained with Pvs25 mouse antiserum and visualized with horseradish peroxidase (HRP)-conjugated anti-mouse IgG secondary antibodies. After imaging, the lower portion of the membrane containing Pvs25 was removed (to reduce signal interference), and the remaining part was probed with mouse β-actin antibodies followed by HRP-conjugated anti-mouse IgG secondary antibodies to visualize β-actin (loading control). Poly(C) RNA served as the negative control; recombinant Pvs25 served as the positive control.

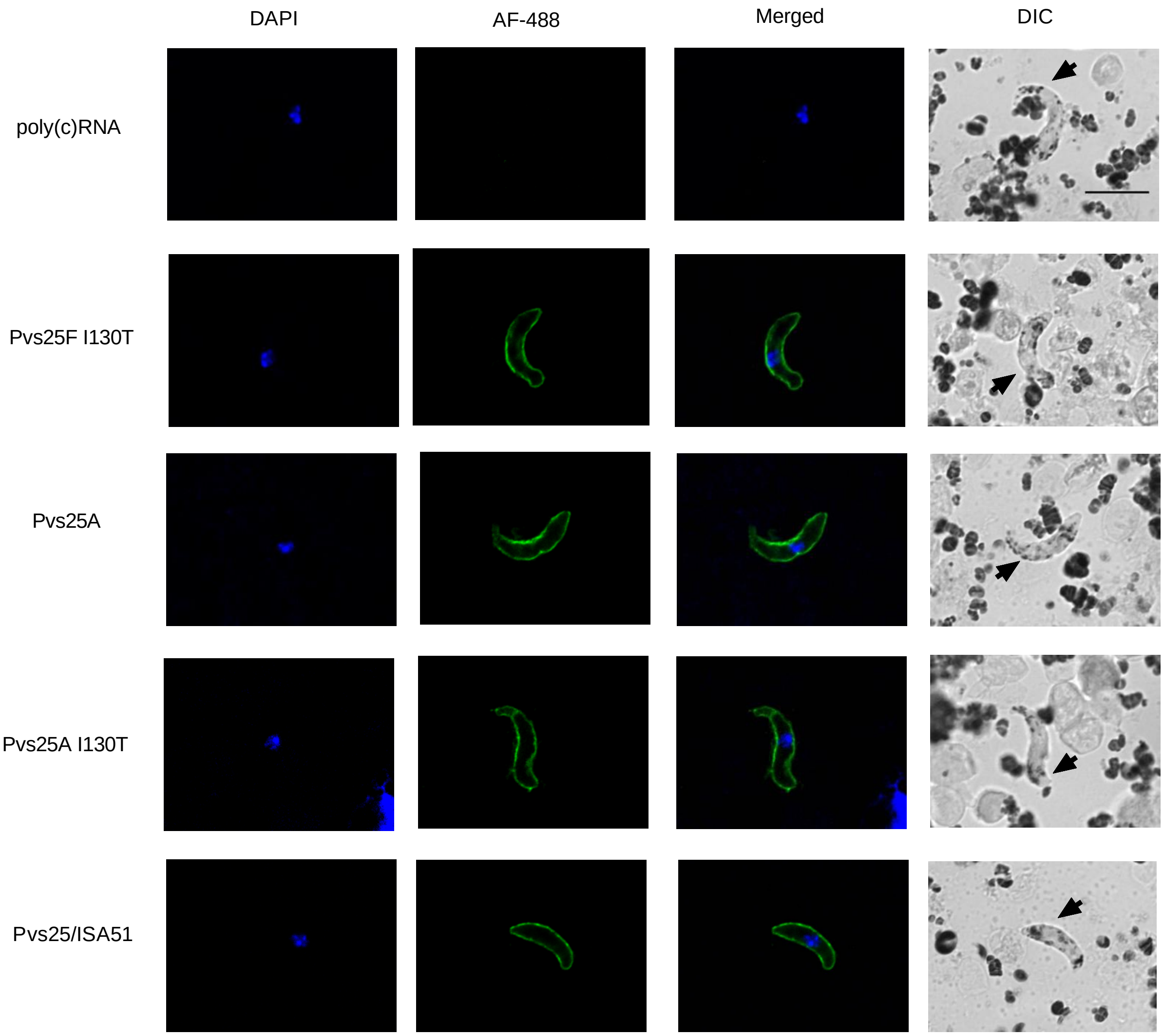

Supplementary Fig. 2 Antibodies induced by Pvs25F I130T, Pvs25A, and Pvs25A I130T mRNA-LNPs and recombinant Pvs25 formulated with Montanide ISA51 (Pvs25/ISA51) recognized the native Pvs25 antigen by IFA. Scale bar indicates 10 µm.

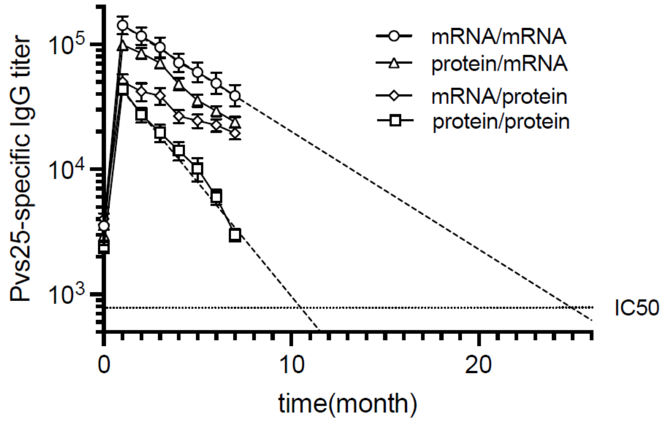

Supplementary Fig. 3 Extrapolation of the antibody decline after the completion of mRNA/mRNA and protein/protein immunizations.
