# Supplementary material for: A Pvs25 mRNA vaccine induces complete and durable transmission-blocking immunity to Plasmodium vivax
Source: NPJ Vaccines. 2023 Dec 14;8:187. doi: 10.1038/s41541-023-00786-9 (PMC10719277; doi:10.1038/s41541-023-00786-9)
Supplement: Supplementary file 2 — Reporting Summary [file 41541_2023_786_MOESM2_ESM.pdf]

nature portfolio

Corresponding author(s): Jetsumon Sattabongkot

Last updated by author(s): Sep 21, 2023

Reporting Summary

Nature Portfolio wishes to improve the reproducibility of the work that we publish. This form provides structure for consistency and transparency in reporting. For further information on Nature Portfolio policies, see our Editorial Policies and the Editorial Policy Checklist.

Statistics

For all statistical analyses, confirm that the following items are present in the figure legend, table legend, main text, or Methods section.

n/a	Confirmed	
☐	☒	The exact sample size (n) for each experimental group/condition, given as a discrete number and unit of measurement
☐	☒	A statement on whether measurements were taken from distinct samples or whether the same sample was measured repeatedly
☐	☒	The statistical test(s) used AND whether they are one- or two-sided<br>*Only common tests should be described solely by name; describe more complex techniques in the Methods section.*
☐	☒	A description of all covariates tested
☐	☒	A description of any assumptions or corrections, such as tests of normality and adjustment for multiple comparisons
☐	☒	A full description of the statistical parameters including central tendency (e.g. means) or other basic estimates (e.g. regression coefficient) AND variation (e.g. standard deviation) or associated estimates of uncertainty (e.g. confidence intervals)
☐	☒	For null hypothesis testing, the test statistic (e.g. F, t, r) with confidence intervals, effect sizes, degrees of freedom and P value noted<br>*Give P values as exact values whenever suitable.*
☒	☐	For Bayesian analysis, information on the choice of priors and Markov chain Monte Carlo settings
☒	☐	For hierarchical and complex designs, identification of the appropriate level for tests and full reporting of outcomes
☒	☐	Estimates of effect sizes (e.g. Cohen's d, Pearson's r), indicating how they were calculated

Our web collection on statistics for biologists contains articles on many of the points above.

Software and code

Policy information about availability of computer code

Data collection	No software was used for data collection.
Data analysis	Statistical analysis was done using Prism 9.

For manuscripts utilizing custom algorithms or software that are central to the research but not yet described in published literature, software must be made available to editors and reviewers. We strongly encourage code deposition in a community repository (e.g. GitHub). See the Nature Portfolio guidelines for submitting code & software for further information.

Data

Policy information about availability of data

All manuscripts must include a data availability statement. This statement should provide the following information, where applicable:

- Accession codes, unique identifiers, or web links for publicly available datasets
- A description of any restrictions on data availability
- For clinical datasets or third party data, please ensure that the statement adheres to our policy

The datasets generated and/or analyzed during the current study are available from the corresponding authors upon reasonable request.

Research involving human participants, their data, or biological material

Policy information about studies with human participants or human data. See also policy information about sex, gender (identity/presentation), and sexual orientation and race, ethnicity and racism.

Reporting on sex and gender	No sex- and gender-based analysis was conducted because sex and gender of malaria patient blood donors are not relevant to the study.
Reporting on race, ethnicity, or other socially relevant groupings	No socially categorization variables were used.
Population characteristics	Clinical infection with Plasmodium vivax
Recruitment	P. vivax malaria patients, age 15 years or older, both male and non-pregnant female were recruited for blood donation for membrane-feeding assays. Only patients with uncomplicated malaria were enrolled. We did not expect that there would be selection bias that would impact the results.
Ethics oversight	Human subject research conducted as part of this study was reviewed and approved by the Ethics Committee of the Faculty of Tropical Medicine, Mahidol University (MUTM-2018-2016-05).

Note that full information on the approval of the study protocol must also be provided in the manuscript.

Field-specific reporting

Please select the one below that is the best fit for your research. If you are not sure, read the appropriate sections before making your selection.

☒ Life sciences ☐ Behavioural & social sciences ☐ Ecological, evolutionary & environmental sciences

For a reference copy of the document with all sections, see nature.com/documents/nr-reporting-summary-flat.pdf

Life sciences study design

All studies must disclose on these points even when the disclosure is negative.

Sample size	Blood samples from 4-5 malaria patients were used to feed mosquitoes in membrane feeding assay for measurement of transmission blocking activity. This choice of sample size was made following standard of the field. It allows sufficient precision for comparing vaccine candidates.
Data exclusions	No data were excluded.
Replication	All immune measurements were made using at least 5 mice to ensure reproducibility.
Randomization	Not applicable.
Blinding	Not applicable

Reporting for specific materials, systems and methods

We require information from authors about some types of materials, experimental systems and methods used in many studies. Here, indicate whether each material, system or method listed is relevant to your study. If you are not sure if a list item applies to your research, read the appropriate section before selecting a response.

Materials & experimental systems

n/a	Involved in the study	
☐	☒	Antibodies
☐	☒	Eukaryotic cell lines
☒	☐	Palaeontology and archaeology
☐	☒	Animals and other organisms
☒	☐	Clinical data
☒	☐	Dual use research of concern
☒	☐	Plants

Methods

n/a	Involved in the study	
☒	☐	ChIP-seq
☒	☐	Flow cytometry
☒	☐	MRI-based neuroimaging

Antibodies

Antibodies used	Mouse anti-β-actin antibodies Abcam ab8226; HRP-conjugated anti-mouse IgG secondary antibodies (Merck Millipore 12-349) for Western blot; HRP-conjugated anti-mouse IgG1 (Abcam ab97240) and IgG2a (Abcam ab97245) for ELISA; Alexa 488-conjugated anti-mouse antibody (Invitrogen A20181) for IFA; anti-mouse CD3 (BioLegend 100306), CD4 (BioLegend 100538), IFN-γ (BioLegend 505826) and IL-2 (BioLegend 503808) antibodies in T cell response measurement;
Validation	For western blot, antibodies react to protein band at the right size and positive in Western blot when using anti-mouse secondary antibodies. For cellular immune response, validation was made by appropriate levels of negative controls in the experiments.

Eukaryotic cell lines

Policy information about cell lines and Sex and Gender in Research

Cell line source(s)	ATCC (American Type Culture Collection)
Authentication	A cell line is provided with a certificate of analysis. Cell identify is verified by morphology.
Mycoplasma contamination	Not tested
Commonly misidentified lines (See ICLAC register)	Not to report

Animals and other research organisms

Policy information about studies involving animals; ARRIVE guidelines recommended for reporting animal research, and Sex and Gender in Research

Laboratory animals	BALB/c mice
Wild animals	The study did not involve wild animals.
Reporting on sex	Female only.
Field-collected samples	The study did not involved animal specimens collected from the field.
Ethics oversight	The study animal protocol was approved by the Institutional Animal Care and Use Committee of the Faculty of Tropical Medicine, Mahidol University.

Note that full information on the approval of the study protocol must also be provided in the manuscript.
